# Fear of heights shapes postural responses to vibration-induced balance perturbation at virtual height

**DOI:** 10.3389/fnhum.2023.1229484

**Published:** 2023-09-12

**Authors:** Diana Bzdúšková, Martin Marko, Zuzana Hirjaková, Igor Riečanský, Jana Kimijanová

**Affiliations:** ^1^Department of Behavioural Neuroscience, Centre of Experimental Medicine, Slovak Academy of Sciences, Bratislava, Slovakia; ^2^Department of Applied Informatics, Faculty of Mathematics, Physics and Informatics, Comenius University in Bratislava, Bratislava, Slovakia; ^3^Department of Psychiatry, Faculty of Medicine, Slovak Medical University in Bratislava, Bratislava, Slovakia

**Keywords:** virtual reality, fear of heights, transient period, muscle vibration, balance control, body sway, visual height intolerance, sensory reweighting

## Abstract

**Introduction:**

Standing upright at height is a challenging situation involving intense threat of balance loss and fall. The ability to maintain balance in such conditions requires properly resolving sensory conflicts and is influenced by fear. To get more insight on the role of fear in balance control at height, we explored the dynamics of postural behavior in the situation of enhanced threat of potential balance loss.

**Methods:**

In 40 young individuals with varying fear of heights, we combined simulated exposure to height in a virtual reality environment with bilateral vibration of tibialis anterior muscles which evokes posture destabilization (the so-called vibration-induced falling).

**Results:**

Under such condition of enhanced postural threat, individuals with intense fear of heights showed stronger stiffening of posture compared with individuals with low fear of heights who react more flexibly and adaptively to posture destabilization. This group difference was evident already at ground level but further increased during virtual height exposure.

**Discussion:**

Our data show that fear of height significantly affects posture adaptation to balance-destabilizing events. Our findings demonstrate that the assessment of postural behavior during threatening situations in the virtual reality environment provides valuable insights into the mechanisms of balance control and may be used to develop novel strategies aimed at prevention of falls.

## Introduction

In everyday life, we need to move and interact with the environment, for which a correct postural control is an essential demand. Maintaining upright stance requires accurate integration of visual, proprioceptive and vestibular sensory information since the sensory signals are continuously reweighted based on their reliability and specificity (Peterka, 2002; Assländer and Peterka, 2014). In research, various challenging situations as postural threats are used to examine how dynamic changes in the environment affect balance control (Brown and Frank, 1997; Carpenter et al., 2004; Davis et al., 2011; Osler et al., 2013; Cleworth et al., 2016; Horslen et al., 2018; Peterson et al., 2018; Cesari et al., 2022). One of the most common postural threats is exposure to height by elevating the surface on which individuals stand. Exposure to height can be simulated in virtual reality (VR) environment and number of studies have already shown that height exposure in VR is in many aspects comparable to real-world height exposure (e.g., Cleworth et al., 2012; Robert et al., 2016; Chiarovano et al., 2017; Peterson et al., 2018). As a noteworthy advantage, VR provides a naturalistic sensory experience in controlled, complex, and easily repeatable environments, allowing individuals to be placed in different situations that they might or need otherwise avoid due to fear or safety restrictions.

Regardless of different situations or different postural threats in real or VR environments, generally, we can distinguish between static and dynamic balance conditions. The static condition refers to quiet standing under stable circumstances of the environment (Macpherson and Horak, 2013), while in the dynamic condition the individual needs to react efficiently to changes of the environment to maintain balance (Paillard, 2019). To create dynamic conditions in research, different types of biomechanical perturbations, sensory perturbations or illusions are usually applied. The biomechanical perturbation involves manipulating the mechanical aspects of a task or environment, directly affecting the body’s physical interactions and requiring adaptations in movement and stability. The sensory perturbation involves modifying or limiting sensory inputs, challenging the central nervous system’s ability to process sensory information and adapt motor responses accordingly (Horak et al., 1997). For instance, proprioception which contributes to the sensory control of balance by updating the estimation of internal body verticality (Hlavacka et al., 1995), can be easily manipulated by selective stimulation. Muscle or tendon vibration generates proprioceptive information which is incongruent with actual body position (Roll et al., 1989). In standing individuals, vibration of lower leg muscles or ankle tendons results in a postural response known as vibration-induced falling (Eklund, 1973), characterized by an involuntary body tilt in the direction of the vibration side. Specifically, vibration of the tibialis anterior muscle causes forward body tilt (Eklund, 1972; Roll et al., 1989), alters the perception of verticality, and aligns the whole body with the tilted subjective postural vertical (Barbieri et al., 2008). Hence, this technique is particularly suitable for experimental sensory perturbation and assessment of postural control.

On the other hand, the balance-relevant proprioceptive inputs can be modulated to fit changes in postural context, such as when threat is increased (Davis et al., 2011). For example, Horslen et al. (2013) demonstrated that postural threat led to an amplification of tendon stretch reflexes (T-reflexes), through an increase in muscle spindle sensitivity. Since the vibratory illusion relies on muscle spindle sensitivity to generate the sensory perturbation, we also need to take into account the increase in muscle spindle sensitivity under conditions of elevated postural threat induced by height, as was previously shown (Davis et al., 2011; Horslen et al., 2013, 2018). Increased spindle sensitivity might serve to facilitate feedback gain in novel or attention-demanding situations, better allowing the body to monitor motor performance (Horslen et al., 2013).

Postural stability, analyzed through postural sway, is a reliable indicator for overall safety status of the postural control system and thus a good indicator of fall risk (Chander et al., 2021). Traditional analysis of the center of pressure yields primary outcome variables from the whole trial to characterize postural control, but less attention has been given to the dynamic postural responses occurring in the transient period, i.e., the time window when the availability of sensory information suddenly changes. The traditional (whole trial) approach masks transient postural behavior (i.e., initial destabilization followed by transition to a more stable, quasi steady-state level). When sensory manipulation (e.g., muscle vibration, visual stimulation) starts or stops, the sudden change in sensory input (i.e., sensory transition) requires sensory reweighting to adjust the weight of each sensory signal to generate corresponding balance motor commands in order to reduce body sway (Assländer and Peterka, 2016). Since postural stability depends on the availability and accuracy of the afferent stimuli, which are integrated by the brain, the time period needed for sensory input integration and incorporation in the postural control is critical (Honeine and Schieppati, 2014). Therefore, studying the transient periods is crucial for understanding adaptation to everyday postural challenges and, potentially, preventing falls which often result in body injuries. Nevertheless, there are only few studies in the general population (excluding studies with ballet dancers, athletes, people after leg injury, etc.) which analyzed in detail the transient periods after a sudden change of sensory input or perturbation. For instance, Reed et al. (2020) reported that transient characteristics of postural sway in parallel stance after closing the eyes differ in younger and older adults. Our own previous research has shown that the postural responses to vibratory stimulation offset can reliably distinguish between the age-related and the pathological changes in patients with Parkinson’s disease (Bzdúšková et al., 2018). Taken together, although the characteristics of body sway in transient periods may provide essential insights into both normal and pathological balance control mechanisms, these characteristics remain poorly understood.

Mounting evidence suggests that psychological factors, such as fear, influence postural control. The emotional states affect the sensorimotor processes underlying balance control and can significantly influence postural stability (Teggi et al., 2019), which is especially evident in individuals with fear of falling (Davis et al., 2009) and fear of heights (Bzdúšková et al., 2022). It has been reported that about 30% of the adult population suffers from visual height intolerance, which reduces the quality of life and causes various constraints such as avoidance of heights (Huppert et al., 2020). Height intolerance is thought to originate from an interaction between the psychological and the physiological factors, such as a discrepancy between the visual, vestibular and somatosensory information used for the postural control (Teggi et al., 2019). We have shown in our recent paper (Bzdúšková et al., 2022) that VR height exposure elicited a complex reaction involving simultaneous correlated changes of the emotional state, autonomic activity and postural balance, which were amplified in individuals with fear of heights. Our results, focusing on posture in static conditions, have indicated that a protective postural adjustment (i.e., stiffening) is an inherent part of a complex threat-related psycho-somatic reaction, which prevents postural destabilization (Carpenter et al., 2001; Raffegeau et al., 2020). Yet a question remained open whether such protective mechanism is also adopted when the postural threat is further enhanced by additional sensory perturbation. Relatedly, it is unclear whether such protective postural reaction is modulated by psychological factors, such as fear of heights.

To address this question, we applied sensory perturbation, i.e., vibration of the anterior muscles of the lower leg to enhance the threat of fall by inducing the forward body lean toward the direction of the threat. Sensory perturbation disrupts the normal sensory feedback and challenges the central nervous system’s ability to integrate and process sensory information, requiring the body to adapt its motor responses based on the altered or limited sensory inputs. The automatic balance responses to perturbations are known to be altered when standing at the edge of a real or virtual elevated platform (Brown and Frank, 1997; Carpenter et al., 2004; Cleworth et al., 2016) and the balance regulation system is sensitive to direction of threat (Forbes et al., 2016). To our best knowledge, there is no study exploring the role of fear of heights in posture control using balance perturbation in a VR environment. In this study, we compared dynamic postural responses to bilateral lower leg muscle vibration during exposure to height in VR in individuals with low and high fear of heights. Our expectation was that the enhanced postural threat (exposure to height combined with vibration-induced falling) will elicit stronger posture stiffening, i.e., decreased amplitude and increased velocity of body sway, in the individuals with high fear of heights as compared to those with low fear of heights.

## Methods

### Subjects and ethics statement

Forty-two healthy young adult volunteers completed the whole protocol. Two individuals showed exceptionally high postural instability and were excluded from statistical analysis as outliers, yielding the final sample of 40 individuals (12 men, 28 women, age 27.2 ± 6.2 years). All participants were free of vestibular, neurological, musculoskeletal or other conditions that may affect balance, all had normal or corrected-to-normal vision and were not repeated or extensive users of VR. Excluded were individuals suffering from mental disorders other than acrophobia (however, no participant reported a history of acrophobia as a clinical diagnosis confirmed by a psychiatrist). Participants’ fear of heights was evaluated using the Visual Acrophobia Test (Bzdúšková et al., 2022), which included 11 pictures showing situations involving heights, and participants rated how anxious they would have felt in the depicted situation using a scale ranging from 1 (no anxiety) to 7 (extreme anxiety). The ratings were summed to form the total VAT score. Pilot studies and available evidence have confirmed that VAT has excellent reliability (Cronbach’s α > 0.95), is tightly related to standard diagnostic criteria for acrophobia (*r* = 0.84), and correlates strongly with other self-report measures assessing fear of heights (*r* > 0.721). The median split of the VAT score was used to divide participants into *low fear (LF)* or *high fear (HF)* groups. The analysis of psychophysiological measures of the stress response during VR exposure to height confirmed that this group attribution was valid (for further details, see Bzdúšková et al., 2022). The experimental protocol for this study was approved by the ethics committee of the Centre of Experimental Medicine, Slovak Academy of Sciences and all participants provided written informed consent with study participation prior to the experiment.

### Experimental setup and VR procedure

**Figure 1:**
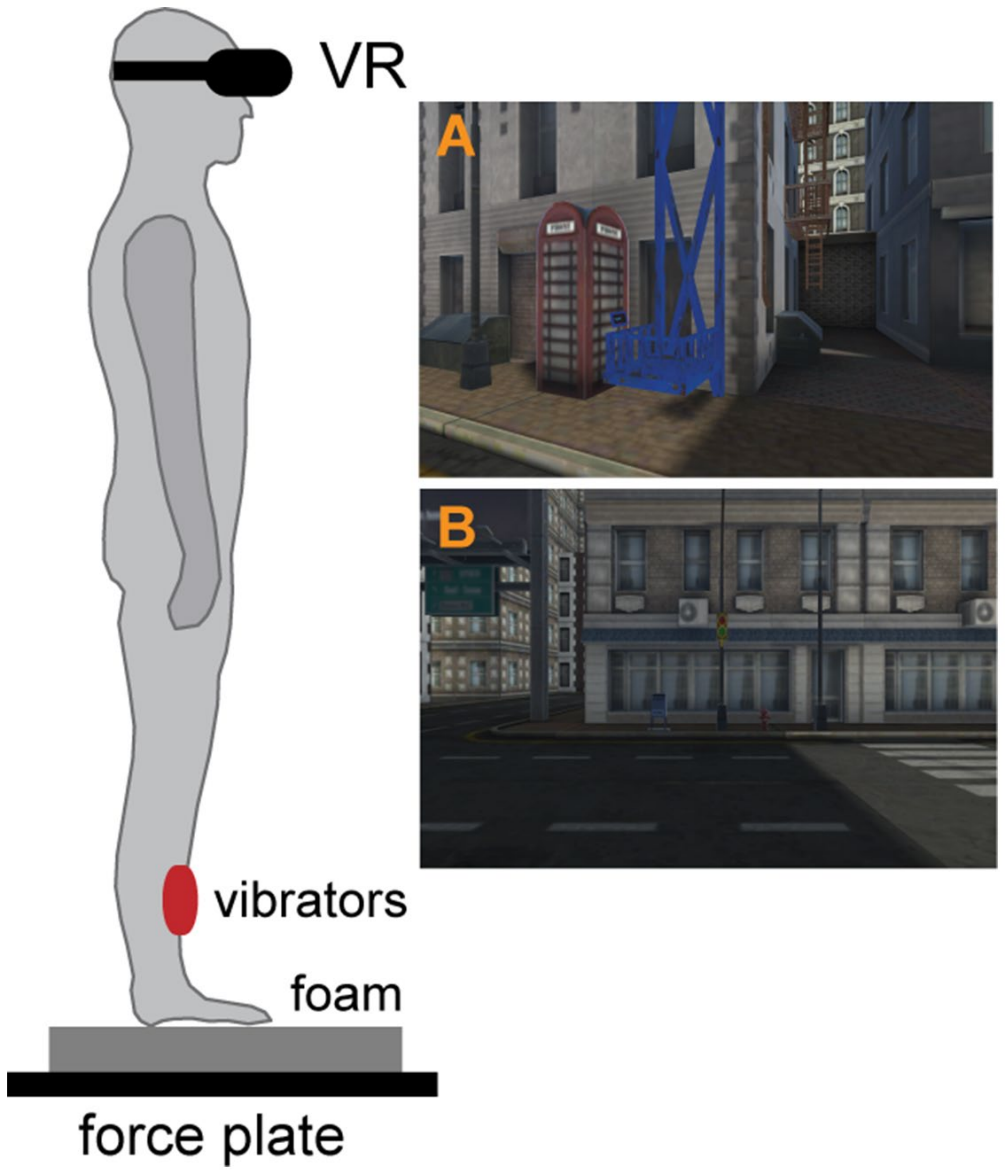
The schematic depiction of the experimental set up including the placement of vibratory units on both tibialis anterior muscles. Example of VR environment: panel **(A)** depicts the virtual elevator and panel **(B)** shows the participant’s view at the ground level.

The analysis of dynamic postural responses was performed as a part of a larger study, which employed VR height exposure. The detailed description of the whole experimental procedure, including study design and psychophysiological measures is provided in Bzdúšková et al. (2022) and here we briefly summarize the key aspects of the procedure. The study had a mixed factorial experimental design and the measurements were carried out within a single session. Virtual reality simulations were implemented and displayed using Oculus Rift (Facebook Inc., California, United States), and involved simulation of an urban environment with an open-air elevator with ~1 m^2^ surface area (see Figures 1A,B). In all *virtual height (VH) stages*, the participants were standing upright in the middle of an in-built force plate and were exposed subsequently with continuous uninterrupted immersion to VR environment to three virtual heights: 0 m (*VH0*), 20 m (*VH20*), and 40 m (*VH40*) in fixed order to maximize the height effect. The elevator was operated/moved by one of the experimenters. At the beginning of the postural measurements, first the data at virtual height 0 m were collected during a stance on a firm support. All subsequent measurements were carried out using a foam pad (50 × 41 × 6 cm, Airex Balance Pad, Switzerland) located on the force plate. The foam pad was used to amplify the destabilizing effect of height and modify the proprioceptive information from feet (Jeka et al., 2004; Simeonov et al., 2005; Chiarovano et al., 2015). After completing the assessments at one height, participants were elevated to the next altitude. At heights of 0 m (*VH0)* and 40 m *(VH40)*, after obtaining static postural measurements, dynamic response to a bilateral vibratory stimulation of lower leg muscles was assessed.

### Postural measures and data analysis

Body sway was quantified by the displacement of the center of foot pressure (CoP) measured by a custom-made in-built force plate (for more details, see Bzdúšková et al., 2022). Participants were instructed to stand quietly, with their feet parallel and at hip width, arms along the body and head in straight position (checked by the experimenter before starting each trial). They were positioned at the center of the virtual elevator with their toes aligned 10 cm away from the front edge of the platform with no possibility to take a step for compensation. To ensure standard and invariant visual input from the simulated scene, all participants were asked to fixate a selected object. At ground level, this object was placed at a distance of ~5 m at the level of eyes. At height, the object was placed at the same distance but below the level of the virtual platform to ensure the perception of height. For safety reasons, one experimenter was standing close to the participant throughout the experiment to provide support in case of postural instability or a tendency to fall.

**Figure 2:**
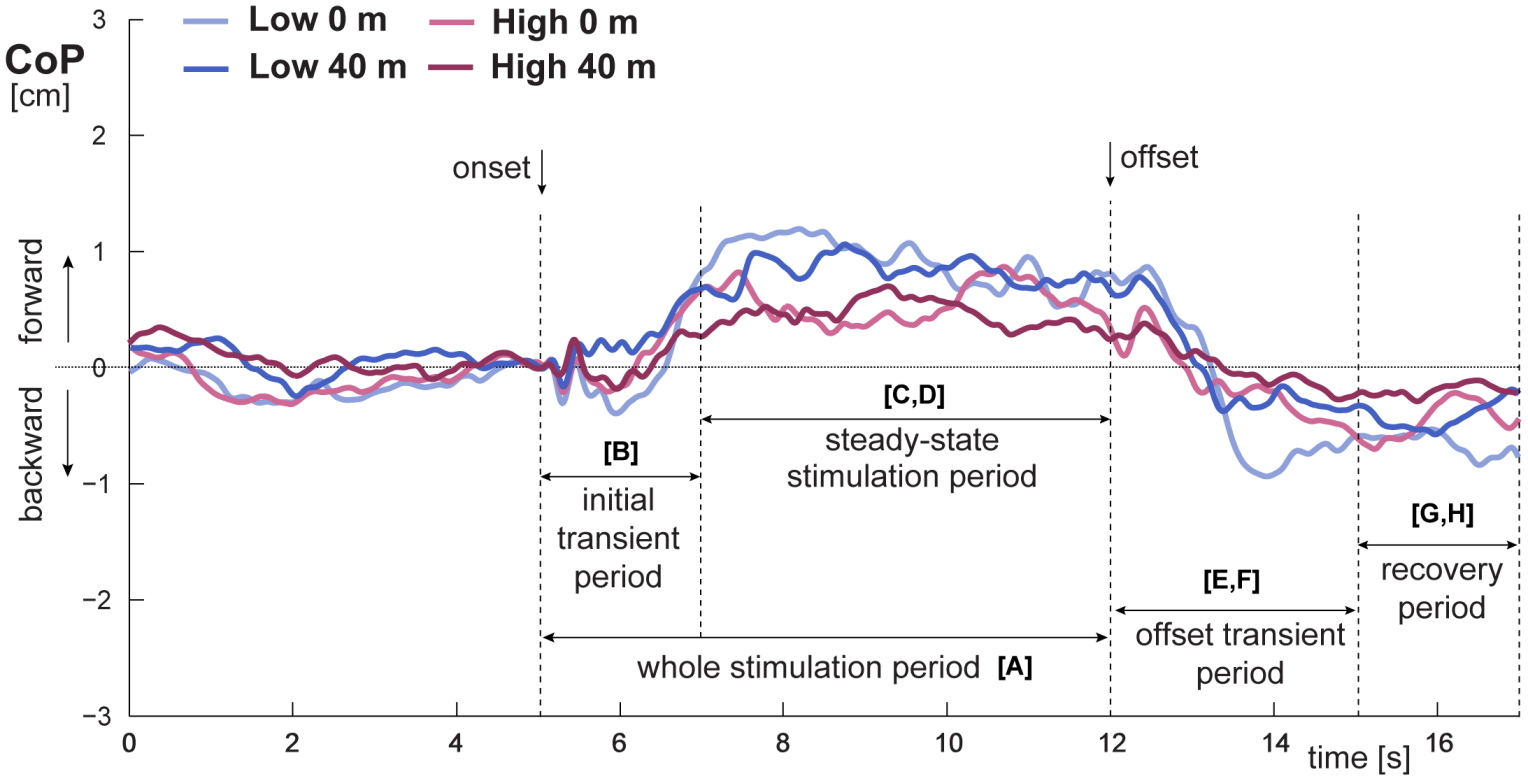
Time series of group mean CoP displacement trajectories in AP direction in the low and high fear groups at virtual height 0 m and 40 m with an illustration of time windows for analysis and parameters calculations. The trajectories are aligned at the moment of vibration onset. The letters in square brackets refer to specific postural parameters which were evaluated in depicted time windows and are displayed as results in Figure 3.

Bilateral proprioceptive stimulation was applied using custom-designed electromechanical vibratory units (DC motor, equipped with small eccentric rotating masses, weighing 230 g, cylindrical in shape, 9 cm long with a diameter of 4.7 cm) which were attached to both tibialis anterior muscle bellies by elastic cuffs. The duration of each dynamic trial was 20 s, consisting of 5 s of quiet stance followed by 7 s of vibration (frequency 80 Hz, amplitude 1 mm) and 8 s of quiet stance (Figure 2). There were two trials at *VH0* and *VH40*. For each participant, CoP responses were averaged across the two trials at each height. First, the individual responses were analyzed and then the summary measures from the two trials of each participant in each height were averaged. Participants´ means were averaged together to obtain group means for both heights. The CoP displacements in anterior–posterior (AP) and medio-lateral (ML) directions were recorded at a sample rate of 100 Hz and processed with a second-order low-pass Butterworth filter with a cut-off frequency of 5 Hz to eliminate low-amplitude measurement noise. Data were analyzed and evaluated with MATLAB^®^ software (Mathworks, Inc., Natick, MA, United States). The mean CoP position in AP direction prior to sensory perturbation (vibration) was calculated from the unbiased CoP signal to determine the magnitude of postural lean in each trial. The mean CoP position was calculated from the first 5 s of trial duration and provides an index of the pre-perturbation posture location. After subtraction the mean CoP position, the bias from the CoP signal was removed at the time point at stimulus onset to provide correct calculations of the postural parameters reflecting responses to the sensory perturbation. For better understanding of postural mechanisms in challenging conditions (i.e., virtual height exposure combined with vibratory stimulation in the direction of facing the threat), specific time windows for analysis were defined (see also Bzdúšková et al., 2018).

**Figure 3:**
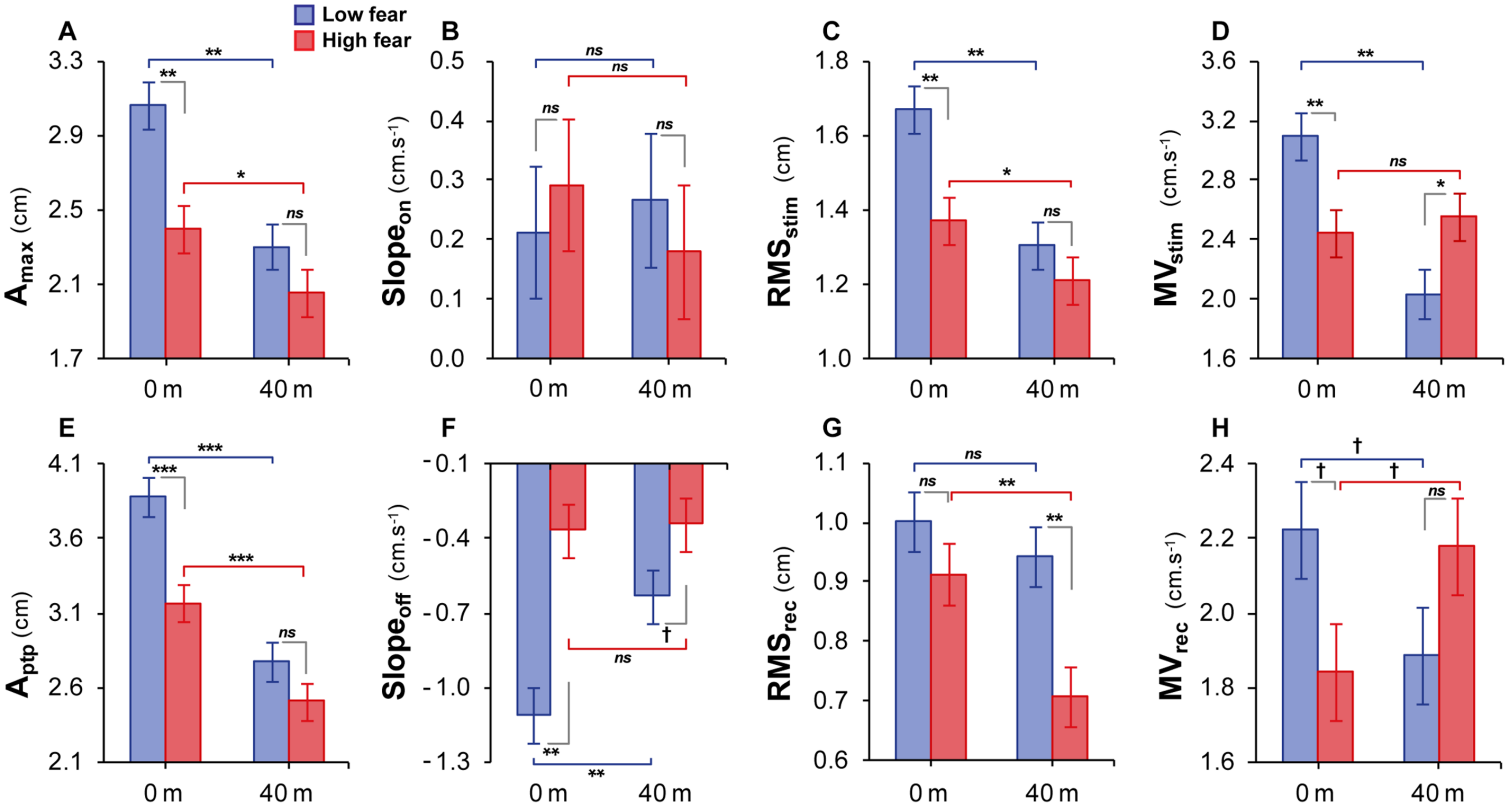
Estimated marginal means ± SEM for all postural parameters in AP direction at 0 m and 40 m virtual heights in the low (blue) and high (red) fear groups: **(A)** A_max_ – the maximal magnitude of forward body tilt in the whole stimulation period; **(B)** Slope_on_ – the maximal CoP velocity in the initial transient period; **(C)** RMS_stim_ – root mean square of CoP in the steady-state stimulation period; **(D)** MV_stim_ – mean velocity of CoP in the steady-state stimulation period; **(E)** A_ptp_ – peak to peak amplitude, range of minimal and maximal final magnitude of body tilt in the offset transient period; **(F)** Slope_off_ – the maximal CoP velocity in the offset transient period; **(G)** RMS_rec_ – root mean square of CoP in the recovery period; **(H)** MV_rec_ – mean velocity of CoP in the recovery period. Significant differences are marked as follows: ^†^*p*  < 0.10, *p < 0.05, **p < 0.01, ***p < 0.001 between the groups by gray color, between the heights in the low fear group by blue and between the heights in the high fear group by red color.

Since the threat of heights is strongest in the facing direction (Adkin and Carpenter, 2018; Zaback et al., 2019, 2021), we applied vibration of tibialis anterior muscles which induces forward body tilt. As a consequence, we analyzed dynamic postural responses only in AP direction. We calculated postural parameters within the following time windows (Figure 2):

1. *whole stimulation period (7 s)* in which we calculated *maximal amplitude (A_max_)* as the maximal magnitude of forward body tilt registered during this period **[A]** (see also Figure 3);
2. *initial transient period (2 s)*, first 2 s immediately after stimulation onset, in which we calculated *slope onset (Slope_on_)* as the initial maximal CoP velocity **[B]**;
3. *steady-state stimulation period (5 s)*, last 5 s of stimulation, in which we assessed for CoP displacement *root mean square (RMS_stim_)* **[C]** and *mean velocity (MV_stim_)* **[D]**;
4. *offset transient period (3 s)*, a three-second period immediately after stimulation offset, in which we calculated *peak-to-peak amplitude (A_ptp_)* as a range of minimal and maximal final magnitude of body tilt **[E]**; and *slope offset (Slope_off_)* as the final maximal CoP velocity **[F]**;
5. *recovery period (2 s)*, a two-second period after offset transient period, in which we assessed for CoP displacement *root mean square (RMS_rec_)* **[G]** and *mean velocity (MV_rec_)* **[H]**.

### Statistical analysis

The data were processed and analyzed in JASP (JASP Team, version 0.14, 2020) and R Studio (RStudio Team, 2019). First, the data were screened for distributional properties and measures showing outlying observations (i.e., values 1.5 interquartile range below Q1 or above Q3) were winsorised using two-sided 20% trimming (separately for each stage and group). Further, the main hypotheses were evaluated using linear mixed effect models (LMEM; lme4 package, Bates et al., 2015) with a fixed within-subject effect *block* (two levels: *VH0* and *VH40*), a fixed between-subject effect group (LF vs. HF), their *interaction*, and a random intercept effect for each participant (default unstructured covariance matrix). All LMEMs were fitted using restricted maximum likelihood and *p*-values were derived with Satterthwaite approximation for degrees of freedom, as these were shown to produce optimal estimates even for smaller samples (Luke, 2017). The semi-partial *R*^2^ was computed to estimate effects sizes for the LMEM analyses.

Furthermore, each LMEM was assessed for four contrasts to test (C1) the difference between the groups at the ground level, (C2) the difference between the groups at height, (C3) the difference between the ground and height (VH0 vs. VH40) in the *low fear* group, and (C4) the difference between the ground and height (VH0 vs. VH40) in the *high fear* group. Statistical significance of the contrasts was adjusted for false discovery rate (Benjamini and Hochberg, 1995) (for more details, see Supplementary material - Supplementary Table 1).

## Results

The bilateral tibialis anterior muscle vibration induced forward body tilt in all participants, regardless of fear of heights. The dynamic postural responses were divided to specific time windows (described in Methods) to assess the effects on postural body sway in more detail (Figure 2). First, we compared responses induced by vibration when standing on the foam vs. firm support at ground level (*VH0*). All participants showed smaller vibration-induced responses when standing on the foam compared to firm support. The LMEMs showed a significant main effect of *surface* on almost all postural parameters except *A_max_* and *Slope_off_* (for more details, see Supplementary material- Supplementary Table 2, Supplementary Figure 1).

Before analysing responses to vibration at virtual height, the mean CoP position was assessed to see if there was a shift in the average location of the CoP during stance prior to perturbation. The LMEMs showed no significant main effect of *group* [*F*_(1, 38)_ = 2.367, *p* = 0.132] or *block* [*F*_(1, 38)_ = 0.384, *p* = 0.539]. The *block x group* interaction was significant [*F*_(1, 38)_ = 5.972, *p* = 0.019]. There was a significant difference in the mean CoP position between the groups at ground level (2.30 ± 0.34 cm for *LF* vs. 3.48 ± 0.34 cm for *HF, p* = 0.014). At virtual height, compared to ground level, the mean CoP position did not significantly change in the *LF* group, while in the *HF* group the mean CoP position shifted significantly closer to the participant’s heels (3.48 ± 0.34 cm at ground level vs. 2.84 ± 0.34 cm at height, *p* = 0.030). In summary, the postural lean before perturbation was not affected by exposure to virtual height in *LF*, but decreased in *HF*. Next, we analyzed responses induced by vibration when standing on the foam at ground level (*VH0*) and at height of 40 m (*VH40*). The results are presented in Table 1, Figures 2, 3. The virtual height combined with vibratory stimulation induced alterations of the CoP displacement compared to ground level in each participant. For the value of the maximal magnitude of forward body tilt (*A_max_*), the LMEMs revealed significant effects of *block* and *group*. *A_max_* was smaller in *HF* than in *LF*, especially at 0 m (Figure 3A). With ascending height, *A_max_* decreased in both groups, which was less visible in *HF*, but the *block x group interaction* did not reach statistical significance. For the initial CoP velocity *(Slope_on_)* immediately after the stimulation onset, we did not find any significant effects of height or fear (Figure 3B).

**Table 1:** Experimental effects induced by exposure to virtual height in combination with vibration: summary of linear mixed effect models for postural measures.

Measure	Effect	df	*F*	*p*	*R* ^2^
A_max_	Block	1, 38	25.025	**<0.001**	0.397
	Group	1, 38	10.845	**0.002**	0.222
	Block × Group	1, 38	3.542	0.068	0.085
Slope_on_	Block	1, 38	0.093	0.762	0.002
	Group	1, 38	<0.001	0.98	<0.001
	Block × Group	1, 38	0.765	0.387	0.020
RMS_stim_	Block	1, 38	28.616	**<0.001**	0.430
	Group	1, 38	6.935	**0.012**	0.154
	Block × Group	1, 38	4.205	**0.047**	0.100
MV_stim_	Block	1, 38	11.494	**0.002**	0.232
	Group	1, 38	0.136	0.715	0.004
	Block × Group	1, 38	17.387	**<0.001**	0.314
A_ptp_	Block	1, 38	63.16	**<0.001**	0.624
	Group	1, 38	12.533	**0.001**	0.248
	Block × Group	1, 38	4.077	0.051	0.097
Slope_off_	Block	1, 38	6.461	**0.015**	0.145
	Group	1, 38	19.963	**<0.001**	0.344
	Block × Group	1, 38	5.303	**0.027**	0.122
RMS_rec_	Block	1, 38	7.146	**0.011**	0.158
	Group	1, 38	9.348	**0.004**	0.197
	Block × Group	1, 38	2.176	0.148	0.054
MV_rec_	Block	1, 38	<0.001	0.98	<0.001
	Group	1, 38	0.096	0.759	0.003
	Block × Group	1, 38	9.575	**0.004**	0.201

The models included the effect of *block* (within-subject factor: *VH0* vs. *VH40*), *group* (between-subject factor: *high* vs. *low fear* of heights) and their *interaction*. All parameters are calculated in anterior–posterior direction. Significant effects are bolded. A_max_ – the maximal magnitude of forward body tilt in the whole stimulation period; Slope_on_ – the maximal CoP velocity in the initial transient period; RMS_stim_ – root mean square of CoP in the steady-state stimulation period; MV_stim_ – mean velocity of CoP in the steady-state stimulation period; A_ptp_ – range of minimal and maximal final magnitude of body tilt in the offset transient period; Slope_off_ – the maximal CoP velocity in the offset transient period; RMS_rec_ – root mean square of CoP in the recovery period; MV_rec_ – mean velocity of CoP in the recovery period.

In the steady-state stimulation period, the LMEMs revealed significant effects of *block, group* and their *interaction* on *RMS_stim_. RMS_stim_* values decreased with ascending height in both groups (Figure 3C). While in 0 m the magnitude of body sway was lower in *HF*, in 40 m the group difference was not significant. For *MV_stim_*, we found significant effects of *block* and *block x group interaction.* With virtual height, the velocity decreased in *LF*, which was not seen in *HF* (Figure 3D).

Within the transient period immediately after the stimulation offset, both parameters, *A_ptp_* (Figure 3E) and *Slope_off_* (Figure 3F), showed significant effects of *block, group* and their *interaction*. In 0 m, there were smaller body tilt and lower *A_ptp_* values for *HF* in comparison to *LF*. At height, individuals in both groups reduced body tilt, in particular *LF*. Velocity of return to the vertical position after vibration offset (*Slope_off_*) was slowed down in *LF* by height exposure. In *HF*, the velocity at ground level was significantly slower when compared to *LF* and was not further decreased by the exposure to height.

In the recovery period, we found significant effects of *block* and *group* for *RMS_rec_*, indicating decreased magnitude of body sway, with the most pronounced reduction at height in *HF*. Furthermore, there was a significant *block x group interaction* for the mean velocity: with ascending virtual height, *MV_rec_* decreased in *LF* but increased in *HF*.

## Discussion

We assessed dynamic postural responses to destabilizing sensory perturbation, specifically to bilateral tibialis anterior muscle vibration, under condition of postural threat at simulated height in individuals with low and high fear of heights. The vibration induced forward body tilt in all participants, but their postural responses were modulated by fear of heights. In particular, individuals with intense fear of heights showed increased stiffening of posture, which was evident already at ground level but further increased during VR exposure to height.

Previous studies of posture control at height focused on static balance and have shown that exposure to height in real and VR environments decreased the magnitude of body sway (Carpenter et al., 2004; Cleworth et al., 2012; Adkin and Carpenter, 2018; Zaback et al., 2019, 2021; Raffegeau et al., 2020; Bzdúšková et al., 2022). In addition, in participants with elevated fear of heights, height exposure increased velocity of body sway and hence stiffening of posture (Wuehr et al., 2019; Bzdúšková et al., 2022), which is a protective reaction that prevents balance destabilization (Adkin et al., 2000; Carpenter et al., 2001; for review see Adkin and Carpenter, 2018). All of these studies suggest that to provide protection against loss of balance, a general strategy is to limit large body movements by stiffening. Therefore, we expected that the enhanced postural threat due to vibration will elicit stiffening, which will be stronger in individuals with high fear of heights.

Prior to sensory perturbation, the mean CoP position as the measure of the pre-perturbation postural lean was analyzed. The mean CoP position indicates the average location of the CoP during stance. Studies which used real height exposure have found that at height, the mean CoP position in AP direction had shifted further away from the edge of the platform (Davis et al., 2009, 2011; Huffman et al., 2009; Naranjo et al., 2015). From these studies, only Davis et al. (2009) included fearful and non-fearful groups like we did and they reported posterior CoP shift in both groups with greater shift in fearful participants. Compared to Davis et al. (2009), who, however, used real height and no foam pad, we observed a smaller postural lean and less sway toward the edge of the platform at height only in *HF* group, but not all participants. On the other hand, Nielsen et al. (2022), who adopted VR, have found no effect of virtual height exposure on the mean CoP position which is consistent with our results in *LF* group. The posterior shift in the mean CoP position at height indicated that the center of pressure moved closer to the heels and further from the edge of the base of support. The *HF* participants thus adopted a more posterior starting position at height and this adjustment led to a more upright posture prior to the sensory perturbation at height. According to Brown and Frank (1997), a more posterior starting position of the center of mass (COM) prior to the perturbation represents a proactive mechanism for COM regulation in postural threat. Adopting an upright posture is an effective proactive adaptation for reducing the risk of falling as it increases the distance of the COM from the forward limit of support and leads to a wider margin of safety (Brown and Frank, 1997). Also, it is worth mentioning the difference in the mean CoP position at ground level between the groups, where the *LF* group showed smaller pre-perturbation postural lean compared to the *HF* group indicating forward shift in the mean CoP position in this group. We assume it could stem from a different postural strategy accommodated by fearful participants at ground, as the forward lean is beneficial in minimizing postural instability (Brown and Frank, 1997). However, this strategy is not appropriate while standing at height, because a forward lean would bring the body closer to the edge of the platform and increase the perceived intensity of danger. From this perspective, *HF* participants appear to rapidly switch between different postural strategies considering height as a threatening situation and they adaptively modulate postural adjustments in order to minimize potential consequences of instability.

Moreover, we note the participants were standing on the foam pad in this experiment. This is an important factor, because the instability of support due to the foam changes the role of proprioceptive information and equilibrium maintenance. As we expected, the combination of foam with vibration evoked smaller dynamic responses in comparison to firm support regardless of perceived fear. The results are in line with other studies showing that the vibration of the leg muscles had a smaller effect on body sway while standing on foam (Kiers et al., 2012) or unstable support (Hatzitaki et al., 2004). Vibration during the stance on foam caused protective stiffening seen by decreased magnitude of body sway (*A_max_*, *RMS_stim_*) as well as increased mean sway velocity (*MV_stim_*) in the *steady-state stimulation period*. When comparing the groups, all analyzed parameters had lower values in the *HF* than in the *LF* group. When standing on foam, as compared to firm support, participants in both groups showed smaller forward vibration-induced body tilt (direction of threat), but the *HF* group showed more stiffened posture and more restricted postural sway. In the *recovery period,* magnitude (*RMS_rec_*) and velocity (*MV_rec_*) of body sway increased in both groups, indicating partial release of the stiffening (see Supplementary material for more detail).

At height, all participants restricted their postural sway during the stimulation period as well as in the offset transient period. In this situation with real possibility of balance loss, the body sway restriction toward the direction of threat, regardless of fear, has apparently a protective aim. When standing at height, limiting postural sway and leaning away from the direction of threat reduces the likelihood of falling over. As pointed out by Zaback et al. (2021), this behavior primarily reflects context-appropriate adaptation to threat rather than the psychological state of fear. On the other hand, the magnitude of body sway was overall smaller in *HF* compared to *LF* participants not only at height but also at ground level, indicating overall stronger restriction of body sway in *HF* individuals. Interestingly, the group differences were actually greater at ground than at height. In other words, the elevated postural threat due to height exposure decreased the difference in postural reaction to sensory perturbation between *HF* and *LF* group. This indicates that postural responses might be influenced by anticipatory fear, which is stronger in *HF* than *LF* individuals. Moreover, the impact of anticipatory fear seems to be stronger than actually perceived fear since group differences in the intensity of perceived distress were in these individuals much greater at height than at ground (as we have previously shown in Bzdúšková et al., 2022). Furthermore, the *HF* individuals showed enhanced effect of support destabilization, i.e., they reacted with increased stiffening when standing on the foam (see Supplementary material). Our results suggest that individuals with increased fear of heights may overestimate the incoming danger, resulting in more stiffened posture. In *LF* individuals, postural control is more adaptive and they react more flexibly to the danger of posture destabilization.

Velocities of CoP displacement at the beginning and the end of the stimulation represent rapid postural adjustments in *transient periods* of sudden change in sensory input. The initial velocity of body sway was similar in both groups and independent of height, indicating that the rapid involuntary forward body lean due to tibialis anterior muscle vibration is a rather invariant reflexive postural response. In contrast, when vibration ceased, return velocity to the vertical position slowed down at height in *LF* individuals. In *HF* individuals, however, return velocity was slow regardless of height (and was even slower in *HF* individuals at ground than in *LF* individuals at height), further indicating the significant impact of anticipatory fear on postural control. Our data show that while initial velocity seems to be intact, the return velocity is sensitive to an individual’s fear of heights. This finding of greater sensitivity of return velocity to detect changes in dynamic conditions resembles that from elderly individuals (Bzdúšková et al., 2018; Reed et al., 2020). In the *recovery period*, the stiffening was present only in the *HF* group, seen as decreased magnitude and increased velocity of body sway. In summary, the sensory transition period after vibration offset requires increased sensory control and it seems that it is also significantly influenced by perceived fear. Also, in the *HF* participants the effect of enhanced threat could persist longer than in people with no or low fear.

The analysis of the response to sensory perturbation (i.e., vibration) yielded detailed characteristics of dynamic postural behavior and the influence of the emotional state on the sensorimotor processes underlying balance control. Postural stability requires continuous reweighting of sensory information from vestibular, visual and somatosensory systems (Peterka, 2002; Assländer and Peterka, 2014), and the exposure to virtual height combined with vibration leads to a sensory conflict where sensory inputs from each system give discordant sensory information. When vibration stops while standing at height, individuals need to redefine the contributions of different sources of sensory information to regulate posture under the condition of enhanced threat. Besides that, the perceived fear could meaningfully affect sensory information. The sensory transient period after vibration onset as well as after vibration offset results in increased postural sway and requires increased sensory control. When the somatosensory information from feet and ankles is altered by vibration, individuals must rely more on the visual and the vestibular systems to maintain balance. As the visual system provides important afferent feedback in the maintenance of postural stability (Horak, 2006; Bronstein, 2019) and is critical for processing threat-related cues (Lelard et al., 2014), one has to consider also the changes of the visual field due to height and a stronger visual dependence in people with height intolerance (Teggi et al., 2019). In particular, we have shown that individuals with fear of heights rely more strongly on visual cues to maintain balance and decreased availability of visual cues at height may result in an exaggerated estimation of the intensity of the danger (Bzdúšková et al., 2022). Also, it is worth mentioning in this context that visual detection thresholds are decreased under arousing conditions (Kim et al., 2017), enhanced threat increases detection accuracy (Vermehren and Carpenter, 2020) and visual-balance reflexes at height could be relatively unchanged or slightly increased (Nielsen et al., 2022).

Furthermore, we also need to consider the effect of fear on the vestibular function. Previous research has shown that vestibular-evoked responses to imposed or self-motion are augmented in fear or increased vigilance (Balaban, 2002) and vestibular gains are increased in threatening conditions (Osler et al., 2013; Horslen et al., 2014; Naranjo et al., 2015; de Melker Worms et al., 2017). As the vestibular system is a significant and important input for balance, altered vestibulospinal function has been proposed as a mediator of postural threat-related changes to balance control (Carpenter et al., 2004). It is possible that fear or anxiety-mediated changes to balance control are affected by altered central processing of vestibular information, as was shown previously (Horslen et al., 2014). Likewise, significant associations between threat-related changes in vestibulospinal reflexes and psycho-physiological parameters (fear and arousal) were previously reported (Naranjo et al., 2015). Also, it seems that fear of falling might differentially affect the feedforward and feedback components of the vestibular-evoked balance response (Osler et al., 2013). Therefore, there is no doubt that fear considerably modifies vestibular reflexes in balance control, but based on our data, we have only indirect evidence to further support claims from above mentioned studies. Nevertheless, we may suppose that increased gain of the vestibular system could also play a role in the differences between *HF* and *LF* participants found in our study.

Although all individuals, regardless of perceived fear, received a sufficiently strong stimulus to minimize sway during enhanced threat, the group differences were augmented at ground pointing to different strategies between groups to deal with the threat. One of the possible explanations could be the threat-related changes in the sensitivity of the muscle spindles. The increased muscle spindle sensitivity under conditions of increased postural threat may serve a functional role in maintaining postural control. For example, the ankle joint stiffening to maintain a tighter control of the COM during quiet stance has been observed in increased postural threat conditions (Carpenter et al., 2001; Davis et al., 2011) and would be potentially facilitated by increased gain of spinal stretch reflexes in the soleus muscles (Horslen et al., 2013). The increased sensitivity might reflect an adaptation strategy to satisfy the conflicting needs to restrict movement when threatened and to maintain a certain amount of sensory information related to postural control. The sensory modalities can adapt to different contexts and those automatic behavioral changes seen with threat may be linked to changes in sensory monitoring of postural control (Horslen et al., 2013). As noted by Horslen et al. (2018), “context-dependent scaling of stretch reflexes forms part of a multisensory tuning process where acquisition and/or processing of balance-relevant sensory information is continuously primed to facilitate feedback control of standing balance in challenging balance scenarios”. Because the muscle spindles are more sensitive to stretch and larger under threatening conditions (Davis et al., 2011; Horslen et al., 2013), it is likely that these changes could contribute to the differences between the *LF* and the *HF* group seen in our study. However, it is also possible that the sensitivity of muscle spindles does not differ between the groups and to clarify this point, electromyography should be adopted in future studies.

Taken together, the threat of height resulted in decreased vibration-induced body tilt in forward direction and restricted postural sway during vibration. The individuals in the *HF* group, however, showed more stiffened posture, which persisted also into the recovery period. In contrast, the *LF* participants reacted primarily with restriction of postural sway in the direction of threat, but did not increase the body sway velocity. Fear thus significantly modulates protective postural adjustments in threatening situations: in the *LF* group the stiffening was partial with alleviation when vibration ceased, whereas in the *HF* group the stiffening was more consistent and elevated throughout the entire trial. It seems that individuals with increased fear of heights may overestimate the incoming danger, resulting in more stiffened posture. Contrary, postural control in low fear individuals is more adaptive and they can react more flexibly to the posture destabilization. We assume that in young adults with high fear, the main and dominating factor is the behavioral effect of the postural threat induced by height, despite disrupting sensory perturbation. Concluding and summarizing, the effect of enhanced postural threat with additional sensory perturbation was obviously affected by intensity of perceived fear. We suggest that fear modulates postural responses in dynamic conditions under threat on multiple levels including fear-related visual dependence, fear-related increased gain of vestibular inputs, increased muscle spindle sensitivity, and more conscious balance control.

## Limitations

Our study used simulation of exposure to height by means of VR and we have no empirical evidence that our findings would be fully equivalent to those from real-world situations of being at height. Yet, the VR environment is immersive and provides a very realistic sensory experience. Importantly, it allows experimental investigations that might not be feasible due to high risk. It is practically impossible to carry out the experiment with individuals standing on a small unprotected platform at the real height of 40 m.

The lack of the assessment of electromyogram to measure activity in lower leg muscles can also be viewed as a limitation of this study. As discussed above, this could provide more insight into the mechanisms mediating the observed effects and should be included in future studies.

Another limitation of the current study is that it included only young healthy participants so that the findings cannot be automatically extrapolated to elderly individuals or patients with health conditions affecting balance control. Finally, given the sample size it is possible that our study has detected only relatively large effects and has not revealed more subtle effects on posture control.

## Conclusion

Regardless of perceived intensity of fear, elevated height together with additional sensory perturbation represent a robust stimulus - postural threat that evokes stiffening in order to minimize body sway and the risk of fall. Participants in both groups swayed less to the edge of the platform and restricted their postural sway despite vibratory stimulation applied in the forward direction, which was a direction of enhanced postural threat. In threatening situations, stiffening of posture is an important reflex to protect from loss of balance and falls. Our results show that this protective reaction is further modulated by fear of heights, i.e., that people with elevated fear maintain more rigid posture and react with more exacerbated stiffening and impaired adaptation to changes in the availability of sensory information and are probably more sensitive to sensory conflicts. On the other hand, the enhanced stiffening could have also negative consequencies. For example, the increased stiffness reported in elderly people interferes with the ability to compensate for balance perturbation (Allum et al., 2002). For now, it is still unclear whether the protective stiffening during threatening situations, which is much stronger in fearful individuals, becomes inappropriate and non-efficient for balance control. More research is required to determine the extent to which excessive stiffening during dynamic balance tasks may adversely affect balance performance and lead to balance instability and, eventually, falls. The study of postural control and sensory integration mechanisms in threatening situations is important for better understanding of the underlying mechanisms and interventions aimed at prevention of falls. Investigations of the relationship between fear and postural responses in dynamic conditions involving sensory perturbation provide valuable information on the postural control mechanisms beyond the basic knowledge coming from the studies of spontaneous body sway.

## Data availability statement

The raw data supporting the conclusions of this article will be made available by the authors, without undue reservation.

## Ethics statement

The studies involving humans were approved by the ethics committee of the Centre of Experimental Medicine, Slovak Academy of Sciences, Slovakia. The studies were conducted in accordance with the local legislation and institutional requirements. The participants provided their written informed consent to participate in this study.

## Author contributions

DB, MM, ZH, IR, and JK: concept and design of the study. DB, MM, ZH, and JK: data collection and analyses. MM conducted the statistical analyses. DB wrote the first draft of the manuscript. MM, ZH, IR, and JK: review and critique. All the authors have taken part in the preparation of this manuscript, have reviewed the results, and have approved the final version of this manuscript.

## Funding

This work was supported by the Slovak grant agency VEGA No. 2/0080/22 and by the Slovak Research and Development Agency under the contract No. APVV-20-0420.

## Conflict of interest

The authors declare that the research was conducted in the absence of any commercial or financial relationships that could be construed as a potential conflict of interest.

## Publisher’s note

All claims expressed in this article are solely those of the authors and do not necessarily represent those of their affiliated organizations, or those of the publisher, the editors and the reviewers. Any product that may be evaluated in this article, or claim that may be made by its manufacturer, is not guaranteed or endorsed by the publisher.
