# Supplementary material for: Fear of heights shapes postural responses to vibration-induced balance perturbation at virtual height
Source: Front Hum Neurosci. 2023 Sep 12;17:1229484. doi: 10.3389/fnhum.2023.1229484 (PMC10523023; doi:10.3389/fnhum.2023.1229484)
Supplement: Supplementary file 1 [file Data_Sheet_1.docx]

***Supplementary Material***

**Fear of heights shapes postural responses to vibration-induced balance perturbation at virtual height**

Diana Bzdúšková, Martin Marko, Zuzana Hirjaková, Igor Riečanský, Jana Kimijanová

***Supplementary Table 1.***

Summary of the *p*-values, which were adjusted to properly control the false discovery rate. C1 refers to the difference between the groups at the ground level, whereas C2 indicates the difference between the groups at height. C3 tests the difference between the ground and height (VH0 vs. VH40) in the *low* *fear* group, whereas C4 indicates the difference between the ground and height (VH0 vs. VH40) in the *high fear* group. † P-values are not adjusted.

A_max_ – the maximal magnitude of forward body tilt in the whole stimulation period; Slope_on_ – the maximal CoP velocity in the initial transient period; RMS_stim_ – root mean square of CoP in the steady-state stimulation period; MV_stim_ – mean velocity of CoP in the steady-state stimulation period; A_ptp_ – range of minimal and maximal final magnitude of body tilt in the offset transient period; Slope_off_ – the maximal CoP velocity in the offset transient period; RMS_rec_ – root mean square of CoP in the recovery period; MV_rec_ – mean velocity of CoP in the recovery period.

|  |  |  |  |  |  |  |
| --- | --- | --- | --- | --- | --- | --- |
| **Measure** | **Contrast** | **Estimate** | **SE** | **z** | **p†** | **p_adj_** |
| A_max_ | C1 | -0.663 | 0.177 | -3.749 | < .001 | 0.002 |
|  | C2 | -0.249 | 0.177 | -1.406 | 0.160 | 0.160 |
|  | C3 | -0.758 | 0.156 | -4.868 | < .001 | 0.002 |
|  | C4 | -0.343 | 0.156 | -2.207 | 0.027 | 0.036 |
|  |  |  |  |  |  |  |
| Slope_on_ | C1 | 0.080 | 0.156 | 0.511 | 0.609 | 0.687 |
|  | C2 | -0.086 | 0.156 | -0.550 | 0.582 | 0.687 |
|  | C3 | 0.054 | 0.134 | 0.402 | 0.687 | 0.687 |
|  | C4 | -0.112 | 0.134 | -0.834 | 0.404 | 0.687 |
|  |  | | | | |  |
| RMS_stim_ | C1 | -0.295 | 0.089 | -3.328 | < .001 | 0.002 |
|  | C2 | -0.094 | 0.089 | -1.063 | 0.288 | 0.288 |
|  | C3 | -0.362 | 0.069 | -5.233 | < .001 | 0.002 |
|  | C4 | -0.162 | 0.069 | -2.333 | 0.020 | 0.027 |
|  |  | | | | |  |
| MV_stim_ | C1 | -0.650 | 0.229 | -2.834 | 0.005 | 0.010 |
|  | C2 | 0.516 | 0.229 | 2.249 | 0.024 | 0.032 |
|  | C3 | -1.057 | 0.198 | -5.346 | < .001 | 0.004 |
|  | C4 | 0.109 | 0.198 | 0.551 | 0.581 | 0.581 |
|  |  | | | | |  |
| A_ptp_ | C1 | -0.716 | 0.178 | -4.026 | < .001 | 0.001 |
|  | C2 | -0.267 | 0.178 | -1.502 | 0.133 | 0.133 |
|  | C3 | -1.108 | 0.157 | -7.047 | < .001 | 0.001 |
|  | C4 | -0.659 | 0.157 | -4.192 | < .001 | 0.001 |
|  |  | | | | |  |
| Slope_off_ | C1 | 0.746 | 0.152 | 4.893 | < .001 | 0.002 |
|  | C2 | 0.290 | 0.152 | 1.902 | 0.057 | 0.076 |
|  | C3 | 0.480 | 0.140 | 3.426 | < .001 | 0.002 |
|  | C4 | 0.024 | 0.140 | 0.169 | 0.866 | 0.866 |
|  |  |  |  |  |  |  |
| RMS_rec_ | C1 | -0.089 | 0.073 | -1.232 | 0.218 | 0.291 |
|  | C2 | -0.235 | 0.073 | -3.243 | 0.001 | 0.004 |
|  | C3 | -0.059 | 0.070 | -0.847 | 0.397 | 0.397 |
|  | C4 | -0.205 | 0.070 | -2.933 | 0.003 | 0.006 |
|  |  | | | | |  |
| MV_rec_ | C1 | -0.381 | 0.184 | -2.071 | 0.038 | 0.051 |
|  | C2 | 0.289 | 0.184 | 1.571 | 0.116 | 0.116 |
|  | C3 | -0.338 | 0.153 | -2.206 | 0.027 | 0.051 |
|  | C4 | 0.333 | 0.153 | 2.170 | 0.030 | 0.051 |
|  |  | | | | |  |
| *Notes: C1 refers to the difference between the groups at the ground level, whereas C2 indicates the difference between the groups at height. C3 tests the difference between the ground and height (VH0 vs. VH40) in the low fear group, whereas C4 indicates the difference between the ground and height (VH0 vs. VH40) in the high fear group. † P-values are not adjusted.* | | | | | | |

***Supplementary Table 2.*** Summary of linear mixed effect models for postural measures on firm and foam surfaces. The models included the effect of *surface* (within-subject factor: firm and foam support on ground level – virtual height 0 m), *group* (between-subject factor: low vs. high fear of heights) and their interaction. All parameters are calculated in anterior-posterior direction. Significant effects are bolded.

A_max_ – the maximal magnitude of forward body tilt in the whole stimulation period; Slope_on_ – the maximal CoP velocity in the initial transient period; RMS_stim_ – root mean square of CoP in the steady-state stimulation period; MV_stim_ – mean velocity of CoP in the steady-state stimulation period; A_ptp_ – range of minimal and maximal final magnitude of body tilt in the offset transient period; Slope_off_ – the maximal CoP velocity in the offset transient period; RMS_rec_ – root mean square of CoP in the recovery period; MV_rec_ – mean velocity of CoP in the recovery period.

| \| **Measure** \| **Effect** \| ***df*** \| ***F*** \| ***p*** \| ***R^2^*** \| \| --- \| --- \| --- \| --- \| --- \| --- \| \| A_max_ \| Surface \| 1, 38 \| 2.134 \| 0.152 \| 0.053 \| \|  \| Group \| 1, 38 \| 7.157 \| **0.011** \| 0.158 \| \|  \| Surface × Group \| 1, 38 \| 0.528 \| 0.472 \| 0.014 \| \|  \|  \|  \|  \|  \|  \| \| Slope_on_ \| Surface \| 1, 38 \| 39.217 \| **< .001** \| 0.508 \| \|  \| Group \| 1, 38 \| 1.071 \| 0.307 \| 0.027 \| \|  \| Surface × Group \| 1, 38 \| 5.347 \| **0.026** \| 0.123 \| \|  \|  \|  \|  \|  \|  \| \| RMS_stim_ \| Surface \| 1, 38 \| 21.754 \| **< .001** \| 0.364 \| \|  \| Group \| 1, 38 \| 2.693 \| 0.109 \| 0.066 \| \|  \| Surface × Group \| 1, 38 \| 0.265 \| 0.609 \| 0.007 \| \|  \|  \|  \|  \|  \|  \| \| MV_stim_ \| Surface \| 1, 38 \| 185.47 \| **< .001** \| 0.830 \| \|  \| Group \| 1, 38 \| 12.246 \| **0.001** \| 0.244 \| \|  \| Surface × Group \| 1, 38 \| 5.194 \| **0.028** \| 0.120 \| \|  \|  \|  \|  \|  \|  \| \| A_ptp_ \| Surface \| 1, 38 \| 29.667 \| **< .001** \| 0.438 \| \|  \| Group \| 1, 38 \| 2.206 \| 0.146 \| 0.055 \| \|  \| Surface × Group \| 1, 38 \| 8.888 \| **0.005** \| 0.190 \| \|  \|  \|  \|  \|  \|  \| \| Slope_off_ \| Surface \| 1, 38 \| 2.704 \| 0.108 \| 0.066 \| \|  \| Group \| 1, 38 \| 5.235 \| **0.028** \| 0.121 \| \|  \| Surface × Group \| 1, 38 \| 19.969 \| **< .001** \| 0.344 \| \|  \|  \|  \|  \|  \|  \| \| RMS_rec_ \| Surface \| 1, 38 \| 67.916 \| < .**001** \| 0.641 \| \|  \| Group \| 1, 38 \| 0.093 \| 0.762 \| 0.002 \| \|  \| Surface × Group \| 1, 38 \| 4.852 \| **0 .031** \| 0.113 \| \|  \|  \|  \|  \|  \|  \| \| MV_rec_ \| Surface \| 1, 38 \| 175.118 \| **< .001** \| 0.822 \| \|  \| Group \| 1, 38 \| 2.827 \| 0.101 \| 0.069 \| \|  \| Surface × Group \| 1, 38 \| 7.192 \| **0 .011** \| 0.159 \| \|  \|  \|  \|  \|  \|  \| |  |  |  |  |  |
| --- | --- | --- | --- | --- | --- | --- | --- | --- | --- | --- | --- | --- | --- | --- | --- | --- | --- | --- | --- | --- | --- | --- | --- | --- | --- | --- | --- | --- | --- | --- | --- | --- | --- | --- | --- | --- | --- | --- | --- | --- | --- | --- | --- | --- | --- | --- | --- | --- | --- | --- | --- | --- | --- | --- | --- | --- | --- | --- | --- | --- | --- | --- | --- | --- | --- | --- | --- | --- | --- | --- | --- | --- | --- | --- | --- | --- | --- | --- | --- | --- | --- | --- | --- | --- | --- | --- | --- | --- | --- | --- | --- | --- | --- | --- | --- | --- | --- | --- | --- | --- | --- | --- | --- | --- | --- | --- | --- | --- | --- | --- | --- | --- | --- | --- | --- | --- | --- | --- | --- | --- | --- | --- | --- | --- | --- | --- | --- | --- | --- | --- | --- | --- | --- | --- | --- | --- | --- | --- | --- | --- | --- | --- | --- | --- | --- | --- | --- | --- | --- | --- | --- | --- | --- | --- | --- | --- | --- | --- | --- | --- | --- | --- | --- | --- | --- | --- | --- | --- | --- | --- | --- | --- | --- | --- | --- | --- | --- | --- | --- | --- | --- | --- | --- | --- | --- | --- | --- | --- | --- | --- | --- | --- | --- | --- | --- | --- | --- | --- | --- | --- | --- | --- | --- |


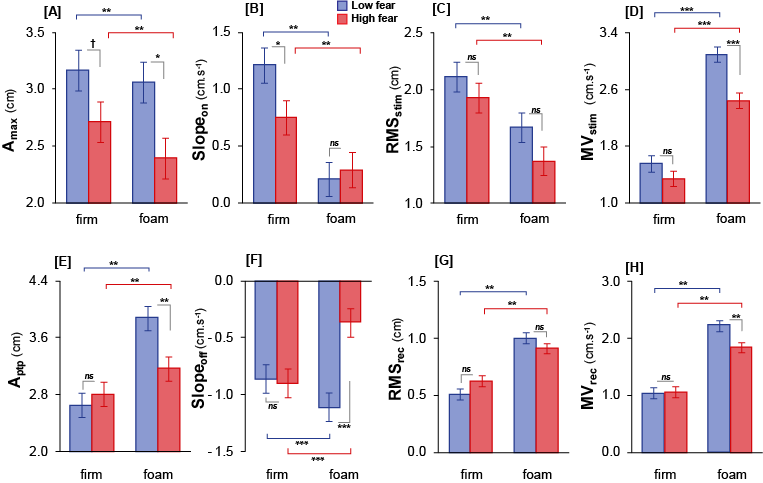


***Supplementary Figure 1.*** Estimated marginal means ± SEM for all postural parameters in AP direction at firm and foam surface in the low (blue) and high (red) fear groups: A_max_ – the maximal magnitude of forward body tilt in the whole stimulation period; Slope_on_ – the maximal CoP velocity in the initial transient period; RMS_stim_ – root mean square of CoP in the steady-state stimulation period; MV_stim_ – mean velocity of CoP in the steady-state stimulation period; A_ptp_ – peak to peak amplitude, range of minimal and maximal final magnitude of body tilt in the offset transient period; Slope_off_ - the maximal CoP velocity in the offset transient period; RMS_rec_ – root mean square of CoP in the recovery period; MV_rec_ – mean velocity of CoP in the recovery period. Significant differences are marked as follows: †*p*<0.10, **p*<0.05, ***p*<0.01, ****p*<0.001 between the groups by grey colour, between the surface in the low fear group by blue and between the surface in the high fear group by red colour.

|  |
| --- |
|  |
|  |
